# Single‐agent gemcitabine in patients with advanced, pre‐treated angiosarcoma: A multicenter, retrospective study

**DOI:** 10.1002/cam4.5147

**Published:** 2022-08-15

**Authors:** Sarah Watson, Benjamin Verret, Stanislas Ropert, Julien Adam, Rastislav Bahleda, Sylvain Briand, Andrea Cavalcanti, Ali N. Chamseddine, Charles Court, Elie Fadel, Matthieu Faron, Leila Haddag‐Miliani, Clémence Henon, Cécile Le Pechoux, Antonin Levy, Olaf Mercier, Carine Ngo, Charles Honoré, Axel Le Cesne, Olivier Mir

**Affiliations:** ^1^ Department of Medical Oncology Curie Institute Paris France; ^2^ Division of Cancer Medicine Gustave Roussy Villejuif France; ^3^ Department of Medical Oncology Antony Private Hospital Antony France; ^4^ Division of Biology and Pathology Gustave Roussy Villejuif France; ^5^ Division of Drug Development (DITEP) Gustave Roussy Villejuif France; ^6^ Department of Orthopedic Surgery, Kremlin‐Bicêtre Teaching Hospital Université Paris‐Saclay Le Kremlin‐Bicêtre France; ^7^ Division of Surgery Gustave Roussy Villejuif France; ^8^ Division of International Patients Care Gustave Roussy Villejuif France; ^9^ Department of Thoracic Surgery, Marie Lannelongue Teaching Hospital Université Paris‐Saclay Le Plessis‐Robinson France; ^10^ Department of Medical Imaging Gustave Roussy Villejuif France; ^11^ Division of Radiation Oncology Gustave Roussy Villejuif France; ^12^ Department of Ambulatory Cancer Care Gustave Roussy Villejuif France

**Keywords:** angiosarcoma, cardiac sarcoma, gemcitabine, sarcoma, soft tissue neoplasms

## Abstract

Gemcitabine has shown clinical activity against angiosarcoma in small series, alone, or combined with taxanes. We aimed to evaluate its activity as a single‐agent in a larger series of patients with advanced angiosarcoma. We retrospectively reviewed the electronic medical records of consecutive adult patients with advanced angiosarcoma treated with single‐agent gemcitabine at our institutions from January 2010 to January 2021. Response was evaluated according to RECIST 1.1, and toxicity was graded according to NCI‐CTC v5.0. 42 patients were identified. 38 patients (90%) had received prior anthracyclines and weekly paclitaxel, and 9 (21%) had received pazopanib. The best tumor response was partial response (PR) in 16 patients (38%), or stable disease (10 patients, 24%). All 8 patients with cardiac angiosarcoma experienced a PR. Median PFS was 5.4 months (95%CI: 3.1–6.5), and median OS was 9.9 months (95%CI: 6.6–13.4). Single‐agent gemcitabine has clinically meaningful activity in advanced, heavily pre‐treated angiosarcoma.

## Graphical abstract

We aimed to evaluate the activity of single‐agent gemcitabine in the largest series of 42 patients with advanced angiosarcoma. The best tumor response was partial response (PR) in 16 patients (38%), or stable disease (10 patients, 24%), median PFS was 5.4 months, and median OS was 9.9 months. Single‐agent gemcitabine has clinically meaningful activity in advanced, heavily pre‐treated angiosarcoma.

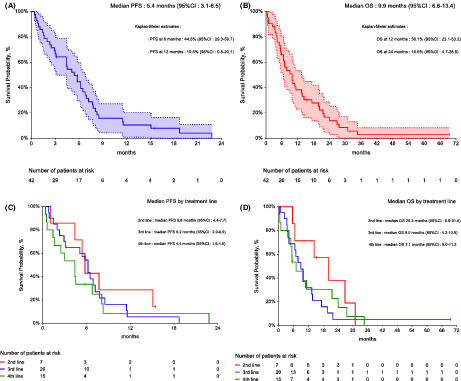

## 1 BACKGROUND

Angiosarcoma are a genetically heterogeneous and aggressive group of mesenchymal tumors that represent 1%–4% of soft‐tissue sarcoma.^1^ Approximately 50% of cases arise in cutaneous sites (in an irradiated field or not), the remaining arising in limbs, trunk, and viscera (including heart and large vessels). Current frontline treatments active against advanced, unresectable angiosarcoma are doxorubicin‐based chemotherapy and weekly paclitaxel,^2^ but systemic treatment options beyond these two key drugs are limited.^1^

Gemcitabine, a cytidine analog with demonstrated efficacy in a broad spectrum of tumors,^3^ has limited clinical activity (as a single‐agent) in unselected soft‐tissue sarcoma.^4^, ^5^, ^6^ Of note, a partial response was observed in one of the three patients with angiosarcoma in a seminal phase 2 trial in soft‐tissue sarcoma.^7^ Thereafter, gemcitabine has shown clinically meaningful activity against advanced angiosarcoma in single cases or small series, as a single agent, or in combination with taxanes.^8^, ^9^, ^10^, ^11^, ^12^ In the largest series reported to date (*n* = 25), a response rate of 68% was reported,^13^ leading to suggest its use as salvage therapy for pre‐treated, advanced angiosarcoma in recent international recommendations.^14^

Moreover, gemcitabine seems to be active in tumors overexpressing ABCB1 (MDR1, formerly “P‐glycoprotein”), a transporter frequently expressed in pre‐treated sarcoma.^15^, ^16^ ABCB1 is also involved in resistance mechanisms to anthracyclines and taxanes and represents a hallmark of the pharmacological concept of multi‐drug resistance.^17^

The aim of this retrospective, multicenter study was to evaluate the clinical activity and safety of single‐agent gemcitabine in adult patients with progressive, advanced angiosarcoma.

## 2 PATIENTS AND METHODS

We retrospectively reviewed the medical records of consecutive patients with advanced angiosarcoma treated with single‐agent gemcitabine in our institutions from January 2010 to January 2021, after ethical approval by the local Institutional Review Boards. Informed consent was obtained from all patients or relatives.

Eligible patients were adults with progressive, metastatic angiosarcoma neither eligible for surgical resection nor for a clinical trial, or having exhausted other conventional chemotherapy regimens. All cases were confirmed by a pathologist from the French network of expert sarcoma pathologists (RRePS). Imaging data were centrally reviewed, and only patients with confirmed tumor progression according to RECIST 1.1^18^ before treatment with gemcitabine were included. Patients with exclusive skin involvement were excluded. Patients and tumor characteristics, previous treatment lines, tumor response, and safety were recorded.

Exclusion criteria included bone angiosarcoma and treatment with gemcitabine in combination with other drugs.

### 2.1 Treatment

Patients received intravenous gemcitabine at a dose of 1000 mg/m^2^ on days 1, 8, and 15 of a 28‐day cycle. Treatment was pursued until tumor progression or major toxicity. Standard laboratory tests were performed weekly. G‐CSF was administered in case of clinically meaningful neutropenia. Tumor evaluation using CT or MRI was performed every 2 cycles or earlier if clinically required.

### 2.2 Evaluation criteria

The primary endpoint was the best tumor response according to RECIST 1.1. Secondary endpoints were: progression‐free survival (PFS), overall survival (OS), and toxicity. PFS was defined as the time from gemcitabine initiation to disease progression, death, or last follow‐up. OS was defined as the time from gemcitabine initiation to death or last follow‐up. Gemcitabine‐related clinical and biological toxicities were retrospectively collected and graded according to the National Cancer Institute Common Terminology Criteria for Adverse Events (NCI‐CTCAE) version 5.0.^19^

### 2.3 Statistical analysis

Median, ranges, and 95% confidence intervals (95% CI) were used to describe patients' characteristics. The NCSS 2020™ software (https://www.ncss.com/) was used for data analysis. The Kaplan–Meier method^19^ and logrank tests were used for survival data analyses.

## 3 RESULTS

### 3.1 Patients' characteristics

Between January 1, 2010, and January 31, 2021, a total of 126 patients with metastatic angiosarcoma were treated at our institutions. Among them, 73 did not receive gemcitabine due to altered PS, progression, or death after weekly paclitaxel and/or doxorubicin (*n* = 41), or patient's preference for oral treatments (*n* = 12). Out of the remaining 53 patients, 11 were excluded (eight received gemcitabine in combination with other cytotoxic agents, and another three were not progressive over 3 ± 0.5 months at the time of gemcitabine initiation).

Overall, 42 patients were eligible for the present analysis. Median age was 52 years (range: 25–90), median PS: 1 (range: 0–2), 36 were women. Patients' baseline characteristics are summarized in Table 1. Ten patients (24%) had locally advanced disease; the median number of metastatic sites was two (range: 1–4) in the remaining 32 patients. All patients but four (*n* = 38, 90%) had received prior anthracyclines (either peri‐operatively or in the metastatic setting) and weekly paclitaxel, and nine (21%) had received pazopanib. Hence, gemcitabine was given as second, third, and fourth line in seven (17%), 20 (47%), and 15 (36%) patients, respectively.

**TABLE 1:** Patients baseline characteristics (*n* = 42)

Characteristic	
Age (years): median (range)	52 (25–90)
Performance status: median (range)	1 (0–2)
Gender: *n* (%)	
Male	6 (14%)
Female	36 (86%)
Primary site: *n* (%)	
Breast/chest wall	12 (29%)
Face/scalp	9 (21%)
Heart	8 (19%)
Other viscera	7 (17%)
Limbs	7 (17%)
Disease extent: *n* (%)	
Locally advanced	10 (24%)
Metastatic	32 (76%)
Number of metastatic sites: median (range)	2 (1–4)
Previous systemic treatments in neoadjuvant and metastatic settings: *n* (%)	
Anthracyclines	38 (90%)
Weekly Paclitaxel	38 (90%)
Ifosfamide	13 (31%)
Pazopanib	9 (21%)
Oral cyclophosphamide	6 (14%)
Dacarbazine	4 (10%)
Vinorelbine	4 (10%)

### 3.2 Treatment disposition

The median duration of treatment was 5.2 months (range: 0.4–18.7). All patients but 9 (who rapidly progressed) received at least two cycles of treatment. One patient with metastatic cardiac angiosarcoma died of a non‐tumor‐related cause (head trauma) while she had an ongoing partial response.

Dose reductions (down to 800 mg/m^2^) were needed in three patients (7%) due to cumulative hematological grade 2–3 toxicities after four cycles. Twenty patients (48%) were able to receive one or more subsequent treatment lines after progression under gemcitabine.

### 3.3 Efficacy

**FIGURE 1:**
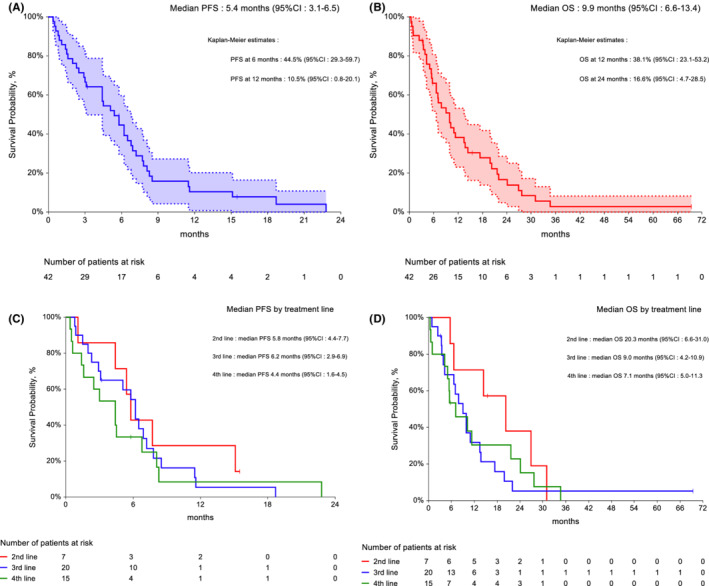
(A) progression‐free survival, *n* = 42; (B) overall survival, *n* = 42. Dotted lines indicate 95% confidence intervals. (C) Impact of previous treatment lines on progression‐free survival. (D) Impact of previous treatment lines on overall survival.

The median follow‐up was 8.5 months (95% CI: 4.7–12.3). The best tumor response was partial response (PR) in 16 patients (38%), or stable disease (10 patients, 24%). All patients with cardiac angiosarcoma experienced a PR, as well as four of 10 patients with angiosarcoma arising in irradiated field, as well as three of the four patients with brain metastases. Median PFS was 5.4 months (95% CI: 3.1–6.5, Figure 1, panel A), and median OS was 9.9 months (95% CI: 6.6–13.4, Figure 1, panel B). No baseline variable (including previous treatment lines) significantly influenced PFS and OS (Figure 1, panel C and panel D).

### 3.4 Toxicity

Toxicity data are summarized in Table S1. Eleven patients (26%) experienced grade 3 toxicity. Grade 3 anemia and thrombocytopenia occurred in three (8%) patients each. Two patients (5%) experienced febrile neutropenia. Two cases (5%) of grade 3 hemolytic and uremic syndrome were observed, both after eight cycles of treatment. No other grade 3 or higher non‐hematological toxicity was observed. No grade 4–5 toxicity was observed.

## 4 DISCUSSION

In this retrospective, multicenter study of 42 patients with advanced, pre‐treated angiosarcoma receiving single‐agent gemcitabine (the largest series reported to date), we observed evidence of significant anti‐tumor activity, along with an acceptable toxicity profile.

The primary aim of this study was to evaluate the anti‐tumor efficacy of single‐agent gemcitabine in second, third, or fourth metastatic line. We were not able to replicate the response rate of 68% reported in a previous series (*n* = 25) from the Italian Rare Cancer Network.^13^ Of note, 7/25 (28%) in the Italian series had previously received doxorubicin and 17/25 (68%) weekly paclitaxel, whereas these rates were 90% and 90% in our series.

The median PFS and OS in the present series (5.4 and 9.9 months, respectively) were lower than those observed in less heavily pre‐treated patients in the Italian series (7 and 17 months, respectively). Hence, our results are not contradictory with those previously reported in a smaller series, and add evidence to the activity of gemcitabine in patients with angiosarcoma pre‐treated with the current standard of care for advanced disease. Notably, the treatment line did not seem to affect PFS, suggesting that gemcitabine might be active even in some multi‐drug resistant tumors, possibly due to its activity in ABCB1‐expressing tumor cells.^16^

In this context, single‐agent gemcitabine appears an attractive option in advanced angiosarcoma, among other options. Currently approved systemic treatment options beyond anthracyclines and weekly paclitaxel are limited to pazopanib^20^ and oral cyclophosphamide (with an activity restricted to angiosarcoma arising in irradiated field, and a potential anti‐angiogenic effect of this schedule^21^).

Anti‐VEGF agents seem a coherent approach given the vascular phenotype of angiosarcoma and the identification of *KDR* (a gene encoding for VEGFR2) activating mutations in up to 26% of cases.^22^ In a retrospective study from EORTC (n = 40,^20^), the use of pazopanib resulted in a response rate of 20% and a median PFS of 3 (95%CI: 2.1–4.4) months. In dedicated phase 2 trials, the use of sorafenib and regorafenib was associated with response rates of 23% and 17.4%, respectively, confirming that oral anti‐VEGF agents exert clinical activity against advanced angiosarcoma.^23^, ^24^ Conversely, the addition of bevacizumab or pazopanib to weekly paclitaxel failed to improve outcomes,^25^ suggesting that combinations involving anti‐VEGF agents should probably be further explored in more selected angiosarcoma subtypes (e.g., angiosarcoma with *KDR* mutations). We have also reported dramatic responses to pazopanib (including one pathological complete response) following treatment with the NOTCH inhibitor crenigacestat,^26^ suggesting that the sequence of treatments in the metastatic setting should be further explored.

Investigational approaches include PD‐1/PD‐L1 inhibition, with several responses reported in small series of patients.^27^ Of note, angiosarcoma arising in the head and neck often exhibits a high tumor mutational burden,^22^ and PD‐1/PD‐L1 inhibition could represent a valuable treatment option in this setting. Other angiosarcoma subtypes vulnerable to PD‐1/PD‐L1 inhibition could be identified using tertiary lymphoid structures (TLS) as a potential predictive biomarker^28^ in an ongoing clinical trial (NCT04095208). Several responses have also been reported under eribulin,^29^ and another phase 2 trial is ongoing (NCT03331250).

Another critical finding of the present series is the activity of gemcitabine in patients with advanced cardiac angiosarcoma, with all eight patients having an objective response. Cardiac angiosarcoma represent a difficult‐to‐treat disease since the primary tumor is often unresectable, metastatic spread (to lungs and brain) is frequent at diagnosis, and the use of cardiotoxic drugs such as anthracyclines might be limited due to altered cardiac function.^30^ Of note, two of three patients with metastatic cardiac angiosarcoma also experienced partial responses in the Italian series.^13^ Given this unexpected response rate on one hand, and previous studies suggesting hENT1 (an efflux transporter for gemcitabine) as a potential biomarker for gemcitabine efficacy^31^, ^32^ on the other hand, we have initiated translational studies to determine whether hENT1 expression could be a targetable alteration in cardiac angiosarcoma.

Overall, our study is limited by its retrospective nature and the lack of translational data on *KDR* mutational status, TLS status, and expression of hENT1. However, we believe that our clinical data strengthen existing recommendations on the role of gemcitabine in the treatment of advanced angiosarcoma.

### 4.1 Implications for clinical care

Gemcitabine is an attractive option in advanced, pre‐treated angiosarcoma due to its schedule of administration (30 min. Intravenous administration, feasible in the ambulatory setting) and a favorable toxicity profile. Single‐agent gemcitabine represents an additional therapeutic option in advanced angiosarcoma adult patients pre‐treated with doxorubicin and/or weekly paclitaxel.

## 5 CONCLUSION

In our experience, single‐agent gemcitabine demonstrated effective palliation along with acceptable toxicity in patients with progressive, advanced angiosarcoma. Our findings confirm that it represents an additional therapeutic option in this population, and its use should be further investigated in specific subpopulations (including cardiac angiosarcoma).

## AUTHORS CONTRIBUTION

All authors have made substantial contributions to acquisition, analysis, and interpretation of data; all authors have been involved in revising the manuscript critically for important intellectual content; all authors have given final approval of the version to be published, and agreed to be accountable for all aspects of the work in ensuring that questions related to the accuracy or integrity of any part of the work are appropriately investigated and resolved.

## CONFLICT OF INTEREST

Dr. Mir has received consultancy fees from Astra‐Zeneca, Blueprint Medicines, Boehringer‐Ingelheim, Bristol‐Myers Squibb, Eli Lilly, Ipsen, Merck Sharpe & Dohme, Pfizer, Roche, Servier, and Vifor Pharma. Dr. Mir is an employee and shareholder of Amgen since February 1, 2022. Dr. Watson has received consultancy fees from Deciphera and Amgen. Dr. Faron has received consultancy fees from HRA Pharma. Dr. Le Péchoux has received consultancy fees from Amgen, Astra‐Zeneca, Eli‐Lilly, Nanobiotix, and Roche. Prof. Court has received consultancy fees from Medtronics and Safeorthopaedic. He is a shareholder of NeuroFrance and SpineGuard. Dr. Le Cesne has received consultancy fees from Amgen, Bayer, Eli‐Lilly, Novartis, Pfizer, and PharmaMar. Other authors have no conflict of interest to disclose.

## Supporting information


Table S1

## Data Availability

Requests for data supporting findings in the manuscript should be made to the corresponding author and will be reviewed individually on a quarterly basis. Data might be shared in the form of aggregate data summaries and via a data transfer agreement. Individual participant‐level raw data containing confidential or identifiable patient information are subject to patient privacy and cannot be shared.

## References

[1] Chen TW , Burns J , Jones RL , Huang PH . Optimal clinical management and the molecular biology of angiosarcomas. Cancers (Basel). 2020;12(11):3321. doi:10.3390/cancers12113321 33182685 PMC7696056

[2] Penel N , Italiano A , Ray‐Coquard I , et al. French sarcoma group (GSF/GETO). Metastatic angiosarcomas: doxorubicin‐based regimens, weekly paclitaxel and metastasectomy significantly improve the outcome. Ann Oncol. 2012;23(2):517‐523. doi:10.1093/annonc/mdr138 21566149

[3] Lund B , Kristjansen PE , Hansen HH . Clinical and preclinical activity of 2′,2′‐difluorodeoxycytidine (gemcitabine). Cancer Treat Rev. 1993;19(1):45‐55. doi:10.1016/0305-7372(93)90026-n 8431926

[4] Okuno S , Edmonson J , Mahoney M , Buckner JC , Frytak S , Galanis E . Phase II trial of gemcitabine in advanced sarcomas. Cancer. 2002;94(12):3225‐3229. doi:10.1002/cncr.10602 12115355

[5] Merimsky O , Meller I , Flusser G , et al. Gemcitabine in soft tissue or bone sarcoma resistant to standard chemotherapy: a phase II study. Cancer Chemother Pharmacol. 2000;45(2):177‐181. doi:10.1007/s002800050027 10663634

[6] Ducoulombier A , Cousin S , Kotecki N , Penel N . Gemcitabine‐based chemotherapy in sarcomas: a systematic review of published trials. Crit Rev Oncol Hematol. 2016;98:73‐80. doi:10.1016/j.critrevonc.2015.10.020 26555460

[7] Patel SR , Gandhi V , Jenkins J , et al. Phase II clinical investigation of gemcitabine in advanced soft tissue sarcomas and window evaluation of dose rate on gemcitabine triphosphate accumulation. J Clin Oncol. 2001;19(15):3483‐3489. doi:10.1200/JCO.2001.19.15.3483 11481354

[8] Kajihara I , Maeda S , Yamada S , et al. Biweekly gemcitabine therapy induces complete remission in cutaneous angiosarcoma resistant to multiple anticancer drugs. J Dermatol. 2015;42(12):1197‐1198. doi:10.1111/1346-8138.13077 26299687

[9] Yonezawa I , Waki M , Tamura Y , et al. Gemcitabine‐based regimen for primary ovarian angiosarcoma with MYC amplification. Curr Oncol. 2014;21(6):e782‐e789. doi:10.3747/co.21.2144 25489268 PMC4257124

[10] Luo ZG , Wang Q , Peng W , Hu XC , Hong XN . Advanced breast angiosarcoma completely responding to gemcitabine‐containing chemotherapy. Breast Care (Basel). 2012;7(5):414‐416. doi:10.1159/000343614 24647783 PMC3518940

[11] Tsubouchi K , Yoshioka H , Ishida T . Significant response to gemcitabine monotherapy in primary pleural epithelioid angiosarcoma. J Thorac Oncol. 2012;7(5):942‐943. doi:10.1097/JTO.0b013e31824feab8 22722799

[12] Bender L , Gantzer J , Somme F , Weingertner N , Kurtz JE . Complete response of a pleural, hepatic, and splenic metastatic primary breast angiosarcoma using gemcitabine monotherapy. JCO Oncol Pract. 2020;16(4):181‐183. doi:10.1200/JOP.19.00609 32069190

[13] Stacchiotti S , Palassini E , Sanfilippo R , et al. Gemcitabine in advanced angiosarcoma: a retrospective case series analysis from the Italian rare cancer network. Ann Oncol. 2012;23(2):501‐508. doi:10.1093/annonc/mdr066 21464156

[14] Gronchi A , Miah AB , Dei Tos AP , et al. Soft tissue and visceral sarcomas: ESMO‐EURACAN‐GENTURIS clinical practice guidelines for diagnosis, treatment and follow‐up. Ann Oncol. 2021 Nov;32:1348‐1365. doi:10.1016/j.annonc.2021.07.006 34303806

[15] Vergier B , Cany L , Bonnet F , Robert J , de Mascarel A , Coindre JM . Expression of MDR1/P glycoprotein in human sarcomas. Br J Cancer. 1993;68(6):1221‐1226. doi:10.1038/bjc.1993.508 7903154 PMC1968648

[16] Bergman AM , Pinedo HM , Talianidis I , et al. Increased sensitivity to gemcitabine of P‐glycoprotein and multidrug resistance‐associated protein‐overexpressing human cancer cell lines. Br J Cancer. 2003;88(12):1963‐1970. doi:10.1038/sj.bjc.6601011 12799644 PMC2741118

[17] Lum BL , Gosland MP , Kaubisch S , Sikic BI . Molecular targets in oncology: implications of the multidrug resistance gene. Pharmacotherapy. 1993;13(2):88‐109. 8097038

[18] Eisenhauer EA , Therasse P , Bogaerts J , et al. New response evaluation criteria in solid tumours: revised RECIST guideline (version 1.1). Eur J Cancer. 2009;45(2):228‐247. doi:10.1016/j.ejca.2008.10.026 https://ctep.cancer.gov/, last accessed on March 18th, 2022 19097774

[19] Altman DG , Bland JM . Time to event (survival) data. BMJ. 1998;317(7156):468‐469. doi:10.1136/bmj.317.7156.468 9703534 PMC1113717

[20] Kollár A , Jones RL , Stacchiotti S , et al. Pazopanib in advanced vascular sarcomas: an EORTC soft tissue and bone sarcoma group (STBSG) retrospective analysis. Acta Oncol. 2017;56(1):88‐92. doi:10.1080/0284186X.2016.1234068 27838944

[21] Mir O , Domont J , Cioffi A , et al. Feasibility of metronomic oral cyclophosphamide plus prednisolone in elderly patients with inoperable or metastatic soft tissue sarcoma. Eur J Cancer. 2011;47(4):515‐519. doi:10.1016/j.ejca.2010.11.025 21251814

[22] Painter CA , Jain E , Tomson BN , et al. The angiosarcoma project: enabling genomic and clinical discoveries in a rare cancer through patient‐ partnered research. Nat Med. 2020;26(2):181‐187. doi:10.1038/s41591-019-0749-z 32042194

[23] Ray‐Coquard I , Italiano A , Bompas E , et al. French sarcoma group (GSF/GETO). Sorafenib for patients with advanced angiosarcoma: a phase II trial from the French sarcoma group (GSF/GETO). Oncologist. 2012;17(2):260‐266. doi:10.1634/theoncologist.2011-0237 22285963 PMC3286175

[24] Agulnik M , Schulte B , Robinson S , et al. An open‐label single‐arm phase II study of regorafenib for the treatment of angiosarcoma. Eur J Cancer. 2021;154:201‐208. doi:10.1016/j.ejca.2021.06.027 34284255

[25] Ray‐Coquard IL , Domont J , Tresch‐Bruneel E , et al. Paclitaxel given once per week with or without bevacizumab in patients with advanced angiosarcoma: a randomized phase II trial. J Clin Oncol. 2015;33(25):2797‐2802. doi:10.1200/JCO.2015.60.8505 26215950

[26] Mir O , Watson S , Massard C , Le Cesne A , Benhadji KA , Soria JC . Sequential treatment with NOTCH inhibitor crenigacestat followed by pazopanib in soft tissue sarcoma patients. Ann Oncol. 2020;31(12):1782‐1784. doi:10.1016/j.annonc.2020.09.003 32941988

[27] Florou V , Rosenberg AE , Wieder E , et al. Angiosarcoma patients treated with immune checkpoint inhibitors: a case series of seven patients from a single institution. J Immunother Cancer. 2019;7(1):213. doi:10.1186/s40425-019-0689-7 31395100 PMC6686562

[28] Petitprez F , de Reyniès A , Keung EZ , et al. B cells are associated with survival and immunotherapy response in sarcoma. Nature. 2020;577(7791):556‐560. doi:10.1038/s41586-019-1906-8 31942077

[29] Fujisawa Y , Fujimura T , Matsushita S , et al. The efficacy of eribulin mesylate for patients with cutaneous angiosarcoma previously treated with taxane: a multicentre prospective observational study. Br J Dermatol. 2020;183(5):831‐839. doi:10.1111/bjd.19042 32198756

[30] Patel SD , Peterson A , Bartczak A , et al. Primary cardiac angiosarcoma ‐ a review. Med Sci Monit. 2014;20:103‐109. doi:10.12659/MSM.889875 24452054 PMC3907509

[31] Maréchal R , Bachet JB , Mackey JR , et al. Levels of gemcitabine transport and metabolism proteins predict survival times of patients treated with gemcitabine for pancreatic adenocarcinoma. Gastroenterology. 2012;143(3):664‐674.e6. doi:10.1053/j.gastro.2012.06.006 22705007

[32] Vincenzi B , Stacchiotti S , Collini P , et al. Human equilibrative nucleoside transporter 1 gene expression is associated with gemcitabine efficacy in advanced leiomyosarcoma and angiosarcoma. Br J Cancer. 2017;117(3):340‐346. doi:10.1038/bjc.2017.187 28641307 PMC5537497

