# Supplementary material for: Single‐agent gemcitabine in patients with advanced, pre‐treated angiosarcoma: A multicenter, retrospective study
Source: Cancer Med. 2022 Aug 15;12(3):3160–6. doi: 10.1002/cam4.5147 (PMC9939156; doi:10.1002/cam4.5147)
Supplement: Supplementary file 1 — Table S1 [file CAM4-12-3160-s001.docx]

**Supplementary Table 1. Adverse events related to weekly gemcitabine in 42 patients with advanced angiosarcoma.**

|  | **Grade 1-2** | **Grade 3** | **Total** |
| --- | --- | --- | --- |
| Fatigue | 16 (38%) | 0 | 16 (38%) |
| Anorexia | 14 (33%) | 0 | 14 (33%) |
| Nausea-Vomiting | 15 (36%) | 0 | 15 (36%) |
| Mucositis | 2 (5%) | 0 | 2 (5%) |
| Alopecia | 3 (7%) | 0 | 3 (7%) |
| Neutropenia | 7 (17%) | 2 (5%) | 9 (21%) |
| Febrile neutropenia | 0 | 2 (5%) | 2 (5%) |
| Anaemia | 7 (17%) | 3 (7%) | 10 (24%) |
| Thrombocytopenia | 10 (24%) | 3 (7%) | 13 (31%) |
| Haemolytic and uremic syndrome | 0 | 2 (5%) | 2 (5%) |
